# Recent HIV infection and associated factors among newly diagnosed HIV cases in the Southwest Ethiopia Regional State: HIV case-based surveillance analysis (2019–2022)

**DOI:** 10.1186/s12879-024-09481-z

**Published:** 2024-06-20

**Authors:** Nigatu Admasu, Abraham Lomboro, Enyew Kebede, Birra Bejiga, Jaleta Bulti, Saro Abdella, Wudinesh Belete, Gemechu Chemeda

**Affiliations:** 1 Ethiopian Field Epidemiology and Laboratory Training Program (EFELTP), Jimma University, Jimma, Ethiopia; 2 Department of Epidemiology, Faculty of Public Health, Jimma University, Jimma, Ethiopia; 3 Southwest Ethiopia Public Health Institute, Tarcha, Ethiopia; 4 Ethiopian Public Health Institute, Addis Ababa, Ethiopia

**Keywords:** HIV case surveillance, Recent HIV infections, Southwest Ethiopia

## Abstract

**Background:**

Globally, there were an estimated 39 million people living with HIV with 1.3 million new HIV infections by the end of 2023. The Sub-Saharan Africa accounted 51% of new HIV infections. HIV case-based Surveillance collects data on newly diagnosed HIV cases, recent HIV infections, and other sentinel events, aiding evidence-based decision making. There is limited evidence on these in Ethiopia. The objective of this study is to determine the incidence proportion of recent infections and associated factors among newly diagnosed HIV cases and their distribution by person, place, and time in the Southwest Ethiopia Regional State.

**Methods:**

A retrospective analysis was conducted on HIV case-based surveillance dataset (July 2019 to June 2022) from the Southwest Ethiopia Regional State. Recent HIV infection is an infection that acquired within the last 12 months as diagnosed by Asante recency test kits. Data were analyzed using SPSS version 26. ArcGIS version 10.8 was used for mapping recent infections. Logistic regression was employed to identify factors associated with recent infections. In multivariable logistic regression analysis, variables with *p*-value < 0.05 and an adjusted odds ratio with 95% confidence interval were considered to declare significant association.

**Results:**

A total of 1,167 newly diagnosed HIV cases (eligible cases) were identified. Among these, 786 (67.3%) recency tests were performed. The mean age of individuals with recent infection was 28.4 years. The proportion of recent infection is 89 (11.3%, 95% CI: 11.2, 11.5%). The highest proportion of recent infection is reported from the West Omo zone (42.9%), whereas 13.2% in Bench Sheko zone. Recent infection is significantly associated with age 15–24 years [AOR = 7.14, 95%CI: 2.89,17.57], age 25–34 years [AOR = 5.34, 95%CI: 2.20,12.94], females [AOR = 2.03, 95%CI: 1.26,3.25], and contact history with the index case [AOR = 0.48, 95%CI: 0.28, 0.83]. The incidence of recent infection increased from 86 (in 2019/20) to 132 (in 2022) recent infections per 1,000 newly diagnosed cases.

**Conclusions:**

Recent HIV infection is a public health concern in the Southwest Ethiopia Regional State with an increasing incidence. Targeted prevention efforts are necessary, especially for females and younger people.

**Supplementary Information:**

The online version contains supplementary material available at 10.1186/s12879-024-09481-z.

## Background

By the end of 2022, there were an estimated 39 million HIV-positive individuals worldwide with 1.3 million newly diagnosed HIV infections [1]. The Sub-Saharan Africa (SSA) had an estimated 660,000 newly diagnosed HIV infections, accounting for 68% of all newly diagnosed HIV infections [2]. The most likely cause of the increase in newly diagnosed HIV infections is the continuation and expansion of risky sexual behaviors, especially among individuals who are HIV-unaware [3]. Recency testing was introduced in Rwanda in 2018 to monitor the age distribution of newly diagnosed HIV positives. Recency testing is a rapid HIV testing that distinguishes between recent and long-term HIV infection using Asante HIV recency test kits [4]. The research conducted in Rwanda on recency testing shows that the proportion of people with newly diagnosed HIV who had recently acquired infections was highest between the ages of 15 and 24 (9.6%) and males over 65 (10.3%), and greater among women (6.7%) than men (5.1%). The percentage of people with newly diagnosed HIV infection decreased, while the number of HIV-positive samples screened for recent infections increased with time [5].

Reducing newly diagnosed HIV infections is essential for HIV epidemic control [6]. It is estimated that 14.5 million people had undiagnosed HIV infection [7]. The National Strategic Plan for HIV 2021–2025 (NSP) of Ethiopia planned to attain HIV epidemic control by 2025, by reducing new HIV infections and AIDS mortality to less than 1 per 10,000 population. To fill this gap, new approaches such as active case finding and recency testing have been incorporated into many national programs with limited HIV-testing resources. According to research done in 2018, HIV case-based surveillance (CBS) and other healthcare databases are increasingly being utilized for public health action, which has the potential to improve the health outcomes of individuals living with HIV [8].

Although Ethiopia has achieved significant success in lowering HIV prevalence and incidence rates, monitoring newly diagnosed HIV infections has proven to be difficult as seen by the slow progress made in reaching the first 90 (diagnosing 90% of HIV positives among the estimated people living with HIV in the community using target testing). By 2030 a new set of targets must be reached, these include: 95-95-95 for treatment: 95% of people living with HIV knowing their HIV status; 95% of people who know their status on treatment; and 95% of people on treatment with suppressed viral loads. The other target was, reducing the annual number of new HIV infections among adults to 200,000 and achieving zero discrimination [9].

This necessitates extra work to identify the groups most responsible for newly diagnosed HIV infections, those who bear the most burden and sick people who would have been overlooked under the current approach [10]. To achieve this, the HIV CBS system was started in Ethiopia in June 2019 which focuse on the identification of recent HIV-1 infections among newly diagnosed cases [11]. Recent HIV infections are infections that are acquired in the past 12 months as detected by Asante HIV recency test kits [11]. Individuals with recent infections exhibit high viral load, an immature and weakened immune response, continued engagement in high-risk behaviors, a heightened likelihood of ongoing transmission (40-60% of transmissions), and an increased capacity to recall their sexual contacts [11, 12]. Identifying recent HIV infections enables rapid initiation of antiretroviral therapy, enhancing health outcomes for patients while reducing transmission risk to others [11].

The HIV CBS is important for monitoring disease trends, detecting newly diagnosed HIV infections, unexpected changes in disease incidence, and assessing the effectiveness of disease prevention programs and policies, all of these depend on an ongoing study of HIV CBS data. Most importantly it helps HIV testing and prevention efforts focus on recent HIV infections that acquired and spread in short period of time. In addition, the system benefits the national HIV program by its endeavors to swiftly respond to subpopulations and sites having high rates of HIV transmission through the detection of recent infections. It is also a key component in the understanding of HIV epidemics control [11, 13]. Furthermore, Ethiopia incorporated newly diagnosed HIV infection in the 36 diseases that are under the national surveillance system and currently receiving reportsweekly from the health facilities to the Ethiopia Public Health Institute through the web-based REDcap (Research Electronic Data Capturing) database system [14].

Therefore, analyzing HIV CBS data provide insights into the epidemiology of recent HIV infections, including trends, demographic characteristics of affected populations, and geographical distribution of cases. This is important for designing effective prevention and intervention strategies tailored to specific populations and regions. In addition, it helps identify risk factors associated with HIV transmission. However, there is limited evidence on what proportion of newly diagnosed HIV infections are recent infections in Ethiopia and particularly in the study area. Therefore, this CBS data analysis aimed to determine the incidence proportion of recent HIV-1 infection among newly diagnosed HIV cases and associated factors and their distribution by person, place, and time in the Southwest Ethiopia Regional State.

## Methods

### Study setting

The study was conducted in the 11 HIV CBS implementing health facilities of the Southwest Ethiopia Regional State, established on November 23, 2021 as the eleventh region of Ethiopia. The region has an estimated total population of 3,311,311as of 2022. It encompasses six administrative zones (Bench Sheko zone, Dawuro zone, Keffa zone, Konta zone, Sheka zone, and West Omo zone), 57 districts, and 16 town administrations and has four capital cities (Bonga, Mizan Teferi, Tarcha, and Tepi towns). It shared borders with the Republic of South Sudan to the South; Gambella Regional State to the West; South Ethiopia Regional State to the East, and Oromia Regional State to the North. There are 13 hospitals and136 health centers in the region. Currently, HIV CBS is implemented in 11 health facilities [Bench sheko zone (Mizan Tepi University Teaching Hospital, Mizan Health Center, Sheko Health Center, and Debrework Health Center), West omo zone (Gabisa Health Center), Sheka zone (Tepi General Hospital, Masha Health Center, and Tepi Health Center), Kaffa zone (Shishoende Health Center and Bonga General Hospital), and Dawuro zone (Tarcha General Hospital)]. These health facilities are currently contributing 95% of the regional HIV cases. These HIV CBS facilities report their weekly surveillance data through an online and real-time reporting software known as REDcap within 15 days of diagnosis of new HIV cases [11].

**Figure:**
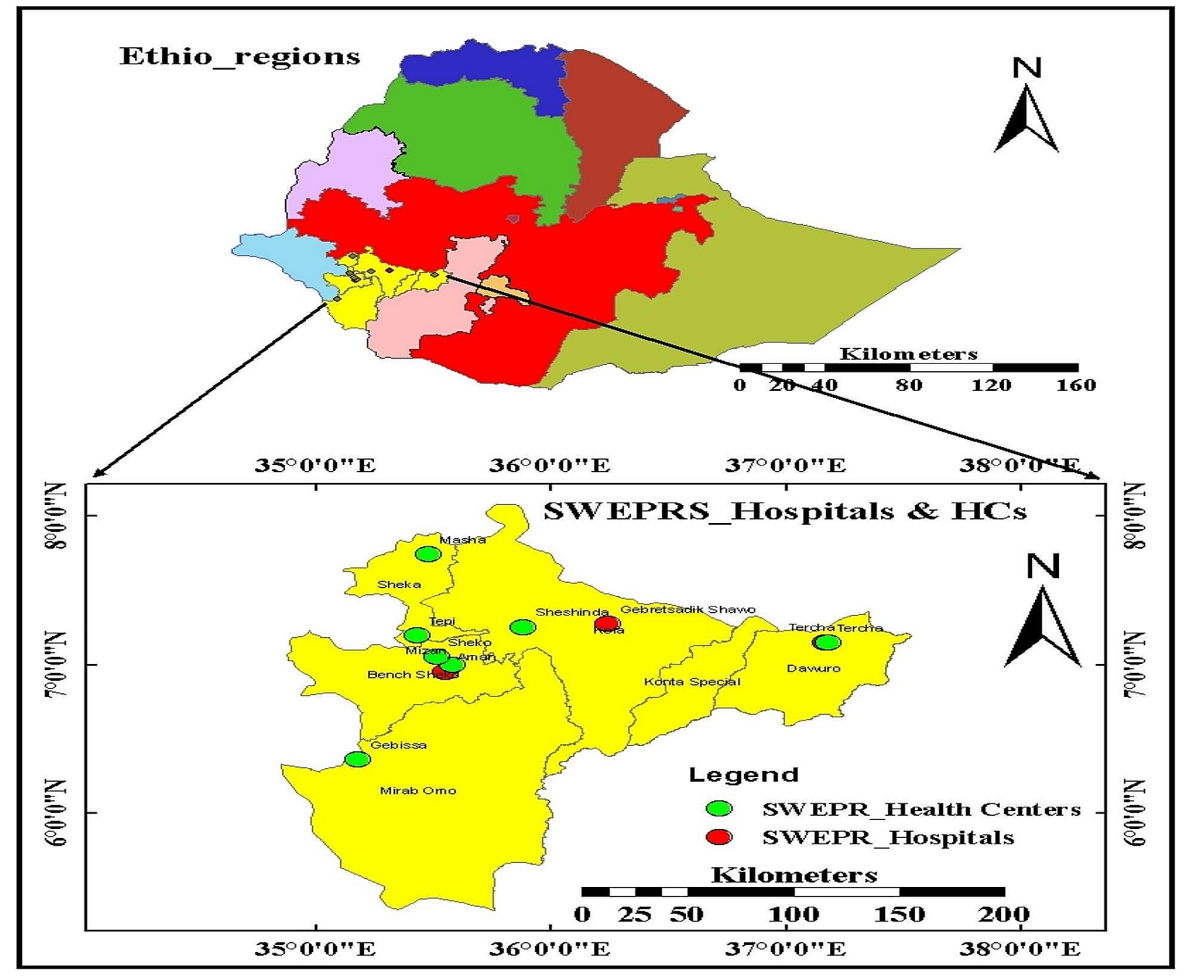
Map of the study area

### Study design, period, and population

A retrospective cross-sectional study was employed using recency testing data collected via the Ethiopian National Research Electronic Data Capture (REDcap) database system (July 2019 to June 2022). For this analysis, there was no specific sampling technique employed since all records during the study period was included. The source population for this study was all newly diagnosed HIV cases. The study population for this study was all newly diagnosed HIV cases who are ≥ 15 years of age during the study period.

### Inclusion and exclusion criteria

The study included all individuals with newly diagnosed HIV infection who are eligible for recency testing (≥ 15 years). Those individuals with newly diagnosed HIV infection and below 15 years old and records with greater than 10% incompleteness on variable information were excluded from the analysis.

### Study variables

Dependent variable: Recency test results (Recent or long-term infection).

Independent variables:

Socio-demographic variables: age, sex, current residency, marital status, current occupation, and educational status.

Risky sexual behavior related factors: contact with index case, have you had sex in the last 12 months, involved in commercial sex practice in the last 12 months, have you injected any illicit drugs to feel good or get high in the last 12 months, and did you receive any blood transfusion/injection/invasive medical procedure in the last 12 months.

Clinical related variables: HIV testing outlets/point of care, WHO staging, and CD4 count.

### Operational definitions

#### HIV case-based surveillance

It is a systematic reporting of newly diagnosed HIV cases and subsequent reporting of their “sentinel events” to a public health authority. Sentinel events include events at the time of diagnosis (e.g. HIV recency test results, first CD4 count, WHO HIV staging) [11]. However, at current stage, the system captures individual level data acquired only within 15 days of HIV diagnosis [4].

#### Newly diagnosed HIV case

An individual who has a newly confirmed diagnosis of HIV infection as per the national HIV diagnosis algorithm [11].

#### Late presenters

Individuals with newly diagnosed HIV cases presenting with CD4 cell counts below 350 cells/mm^3^) at the time of recency testing.

### Asante HIV-1 rapid recency assay

The Asante recency test kit is a rapid point-of-care test used to differentiate between recent and long-term HIV infections [4, 15]. The test strip comprises materials which, in combination, can detect HIV antibodies when a blood, serum, or plasma sample containing HIV antibodies is added to the sample buffer tube. It works based on the principle of avidity, which is the binding strength of the HIV-1 antibodies present in an infected individual’s blood. Following HIV-1 infection, the immune system initially produces low-avidity HIV-1 antibodies, and over time, it matures and produces high-avidity HIV-1 antibodies. This assay is in a rapid lateral flow format and can be used at the point-of-care level [16]. It is reported that the test kit had a high sensitivity (99.1%) and specificity (98.9%) [17].

It is a commercially available kit (Asante recency test R by Sedia Biosciences in Beaverton, Oregon, USA), offers quick results within 20 min. The test comprises three lines: the Control line (C line), the HIV diagnostic verification line (V line), and a third line indicating long-term infection (LT line). Interpretation of results is as follows: presence of only the Control line (C line) indicates HIV-negative status, two lines (C and V lines) suggest recent infection, and three lines (C, V, and LT lines) indicate long-term infection [17]. In Ethiopia, the target population for the recency test includes individuals aged 15 years and above with newly diagnosed HIV-1 infection [11].

#### Recent HIV infection

HIV infection acquired within the last 12 months as identified by Asante HIV recency test kits or it is confirmed as a newly identified HIV infected case and tested positive for recent infection using HIV recency assay [11].

#### Long-term infection

HIV infection which is likely acquired before 12 months or longer prior to the recency test date [4, 18].

#### HIV index case

A person who initially is diagnosed to have HIV infection and serve as a contact person to identify and investigate his/her sexual/biological contacts and risk network [4].

### Data collection tool and procedures

A structured data extraction tool adapted from the Ethiopian guideline on National HIV case report form for newly diagnosed HIV-positive individuals was used [11]. Based on the National guideline, the national HIV CBS advisory committee of the Ethiopian Public Health Institute (EPHI) was communicated to access variables required for this data analysis. The EPHI provided the HIV CBS data after extracting the National REDcap database system. The data extraction was done by experts (data managers) at the EPHI, based on the requested variables.

The data analysis covered the data reported from July 2019 to June 2022. The variables requested included Socio-demographic variables (age, sex, current residency, marital status, current occupation, educational status); Risky sexual behavior related factors (contact with index case, have you had sex in the last 12 months, involved in commercial sex practice in the last 12 months, have you injected any illicit drugs to feel good or get high in the last 12 months, and did you receive any blood transfusion/injection/invasive medical procedure in the last 12 months); Clinical related variables (HIV testing outlets/point of care, WHO staging, and CD4 count).

### Data quality management

The exported data from the REDcap database was checked for completeness and consistency before analysis using Microsoft excel 19. HIV records with more than 10% of the information missing were excluded from the analysis due to the concern about the ability to accurately interpret the data and to be confident that the available data are representative [19]. At the health facility level, the data were recorded by health professionals experienced in HIV CBS and data were extracted by expert data managers at EPHI. Moreover, three years (2019/20-2022) regional newly diagnosed HIV infection reports from the National Demographic Health Information System were cross-checked with the exported data.

### Data processing and analysis

The data was cleaned, coded, and exported to SPSS version 26 for analysis. ArcGIS version 10.8 software was used for mapping the distribution of recent HIV infections. Descriptive statistics such as frequency, percentage, and mean were computed to describe the study participants and the outcome variable. The outcome variable was categorized as recent and long-term infection. Time trends were examined through logistic regression, with recent infection as dependent variable and the number of diagnosed cases per year as the independent variable.

The Hosmer-Lemeshow goodness of fit test was done and indicated that the model fitted with the data(*P* = 0.725). Variables with a *P*-value < 0.25 in the bivariate logistic regression analysis were selected as candidate. In multivariable analysis, variables with *P*-value < 0.05 and an adjusted odds ratio with respective 95% confidence interval were used to identify statistically significant factors associated with recent HIV infection.

### Handling missing variables data

The amount of missing data for the variables was significant, with 29% missing for WHO clinical HIV staging and 60% for CD4 count. The major reported reasons for the missing data were that the HIV CBS system currently prioritizes the reporting of recent HIV infections and newly diagnosed HIV cases. Consequently, data on sentinel events such as ART initiation, CD4 count, WHO HIV clinical staging, viral load count, and others have been given less attention by health professionals during individual level data recording. This has led to substantial data incompleteness for the CD4 and WHO HIV clinical staging variables in this study. To address the issue of missing data and ensure the accuracy of data interpretation, records with more than 10% missing information were excluded from the analysis [19].

## Results

### Socio-demographic, clinical, and risky behavior related characteristics of study participants

**Fig. 1:**
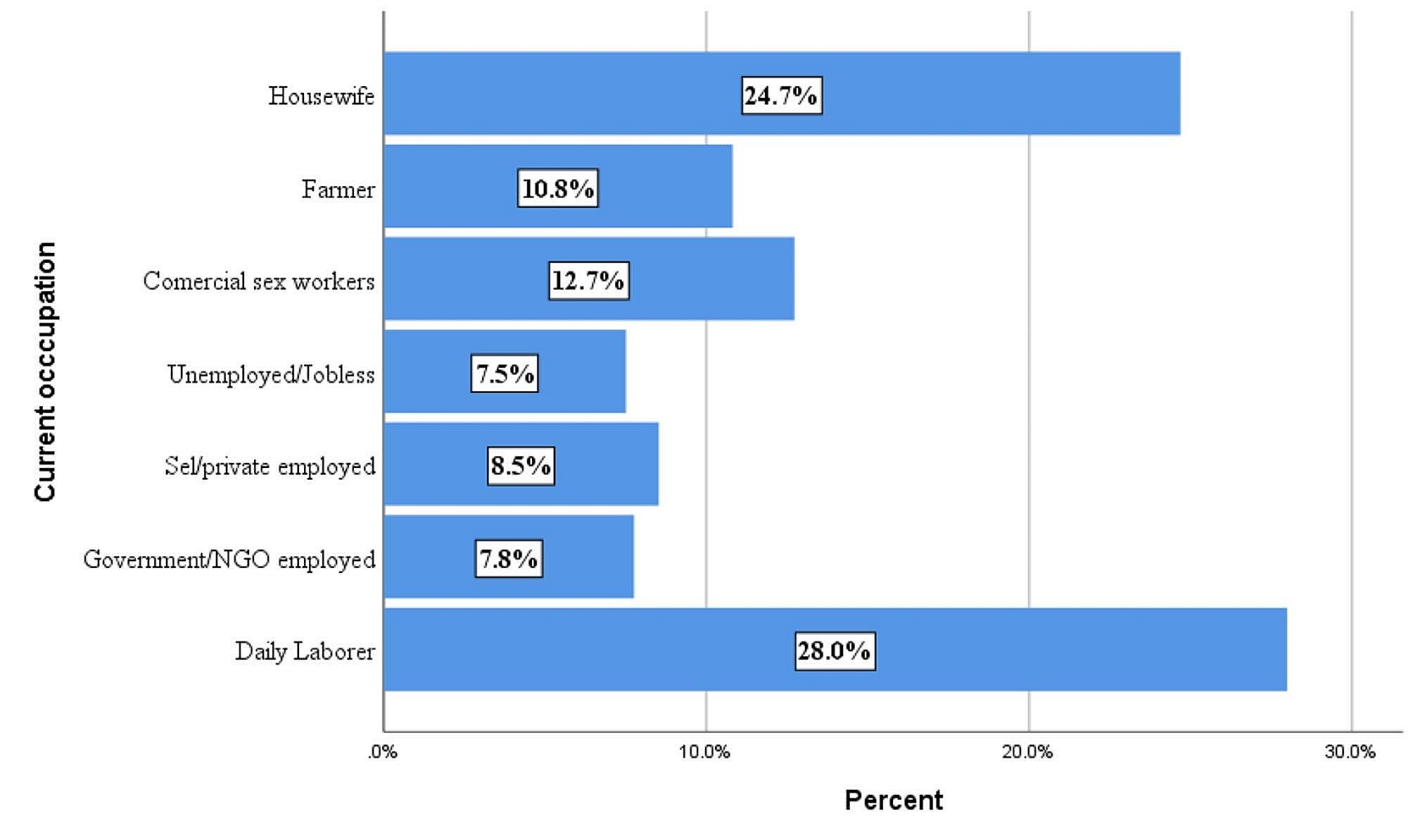
Proportion of newly diagnosed HIV cases among tested for recency by occupation, Southwest Ethiopia Regional State, 2022

During the study period, a total of 1,167 newly diagnosed HIV cases (eligible for recency testing) were reported from 11 health facilities in the region. Of these, recency testing was performed for 786 (67.3%) and 786 results were reported to be valid (the test kit read both recent and long-term infections correctly). The primary reasons for missed recency testing were stockouts of test kits and a lack of attention from healthcare professionals to conduct the recency testing. The mean age of individuals at the time of recency testing was 28.4 years (± 9.17 standard deviation), ranging from 15 to 76 years, with the majority (74%) between the age of 15 and 34. During recency testing, females accounted for 65% and 26.2% were not married or cohabiting. Additionally, 17.4% had no formal education (Table 1). In terms of occupation, 28% of individuals reported working as daily laborers (Fig. 1).

**Table 1:** Socio-demographic, clinical, and risky behavior related characteristics of study participants, Southwest Ethiopia Regional State, 2022 (*N* = 786)

Study variables	Category	Recency test results				Total	
		Recent		Long term			
		N	%	N	%	N	%
Age in years	15–24	40	15.9%	211	84.1%	251	31.9%
	25–34	43	13.0%	288	87.0%	331	42.1%
	>=35	6	2.9%	198	97.1%	204	26.0%
Sex	Female	49	9.6%	462	90.4%	511	65.0%
	Male	40	14.5%	235	85.5%	275	35.0%
Current residence	Shelter	0	0.0%	181	100.0%	181	23.0%
	Prison	23	14.4%	137	85.6%	160	20.4%
	Homeless	2	8.3%	22	91.7%	24	3.1%
	House/apartment	64	15.2%	357	84.8%	421	53.6%
Marital status	Not married/cohabiting	30	14.6%	176	85.4%	206	26.2%
	Divorced/widowed	19	9.9%	173	90.1%	192	24.4%
	Married	40	10.3%	348	89.7%	388	49.4%
Current occupation	Daily Laborers	24	10.9%	196	89.1%	220	28.0%
	Farmers	8	9.4%	77	90.6%	85	10.8%
	Commercial sex workers	13	13.0%	87	87.0%	100	12.7%
	Governmental/non-governmental organization	12	19.7%	49	80.3%	61	7.8%
	Self/private business employed	5	7.5%	62	92.5%	67	8.5%
	Unemployed/jobless	9	15.3%	50	84.7%	59	7.5%
	Housewife	18	9.3%	176	90.7%	194	24.7%
Educational status	Primary	67	12.9%	454	87.1%	521	66.3%
	Secondary and above	5	3.9%	123	96.1%	128	16.3%
	No formal education	17	12.4%	120	87.6%	137	17.4%
Contact with index case	Yes	60	9.8%	552	90.2%	612	77.9%
	No	29	16.7%	145	83.3%	174	22.1%
In the last 12 months, have you had sex?	Yes	46	12.8%	312	87.2%	358	45.5%
	No	43	10.0%	385	90.0%	428	54.5%
In the last 12 months, commercial sex practice?	Yes	22	15.3%	122	84.7%	144	18.3%
	No	67	10.4%	575	89.6%	642	81.7%
In the last 12 months, have you injected any illicit drugs to feel good or get high?	Yes	3	23.1%	10	76.9%	13	1.7%
	No	86	11.1%	687	88.9%	773	98.3%
In the last 12 months, did you receive any blood transfusion/injection/invasive medical procedure?	Yes	2	18.2%	9	81.8%	11	1.4%
	No	87	11.2%	688	88.8%	775	98.6%
Zone of current residency	Bench sheko	68	13.2%	449	86.8%	517	65.8%
	Dawuro	2	10.5%	17	89.5%	19	2.4%
	Kaffa	5	4.8%	99	95.2%	104	13.2%
	Sheka	11	7.9%	128	92.1%	139	17.7%
	West omo	3	42.9%	4	57.1%	7	0.9%
Recent HIV diagnosis year	2019/20	23	15.9%	122	84.1%	145	34.1%
	2021	34	9.0%	342	91.0%	376	33.2%
	2022	32	12.1%	233	87.9%	265	32.7%
WHO HIV clinical stage (*N* = 558)	Stage I/II	25	16.9%	123	83.1%	148	26.5%
	Stage III/IV	47	15.9%	249	84.1%	296	53.0%
	Not recorded	17	14.9%	97	85.1%	114	20.4%
CD4 count (*N* = 317)	< 200	35	38.9%	55	61.1%	90	28.4%
	200–349	23	33.8%	45	66.2%	68	21.5%
	350–500	17	28.8%	42	71.2%	59	18.6%
	> 500	14	14.0%	86	86.0%	100	31.5%

Initial CD4 count was performed for 317 (27%) individuals with newly diagnosed HIV infections. Among those with available CD4 cell count information at the time of diagnosis, 158 (49.8%) were late presenters, with CD4 counts below 350 cells per mm3. Of these late presenters, 90 (28%) had advanced HIV infection (CD4 counts less than 200 cells per mm3). Late presentation was more common among females compared to males, with 110 (69.6%) late presenters being female and 48 (30.4%) being male. The mean CD4 count among individuals with newly diagnosed HIV infections was 406.52 cells/µl. Additionally, more than half (53%) of individuals with newly diagnosed HIV cases presented with WHO clinical stage III or IV at the time of recency testing (Table 1).

### Recent HIV infection

**Fig. 2:**
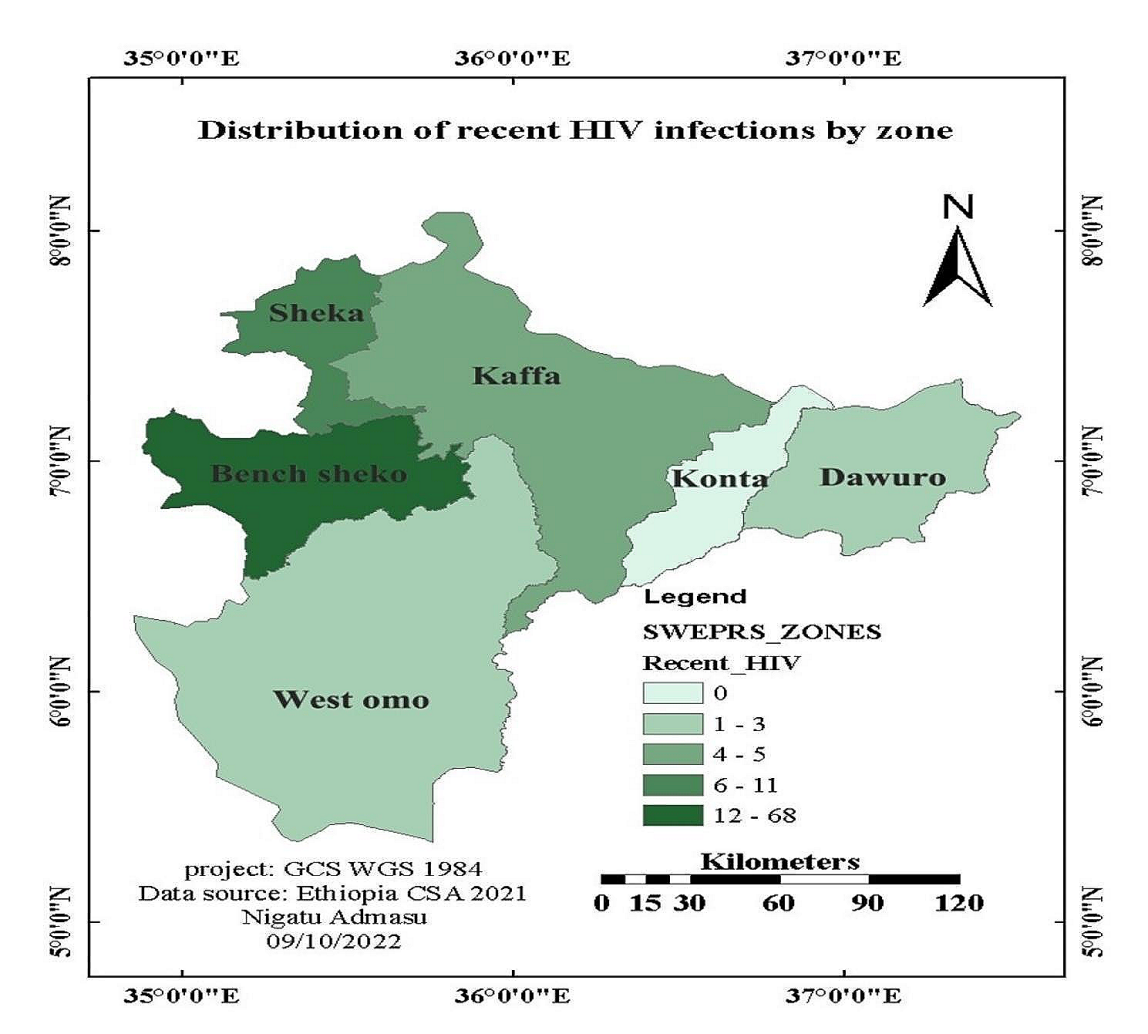
Geographic distribution of recent HIV infections in the Southwest Ethiopia Regional State (*N* = 89), 2022

**Fig. 3:**
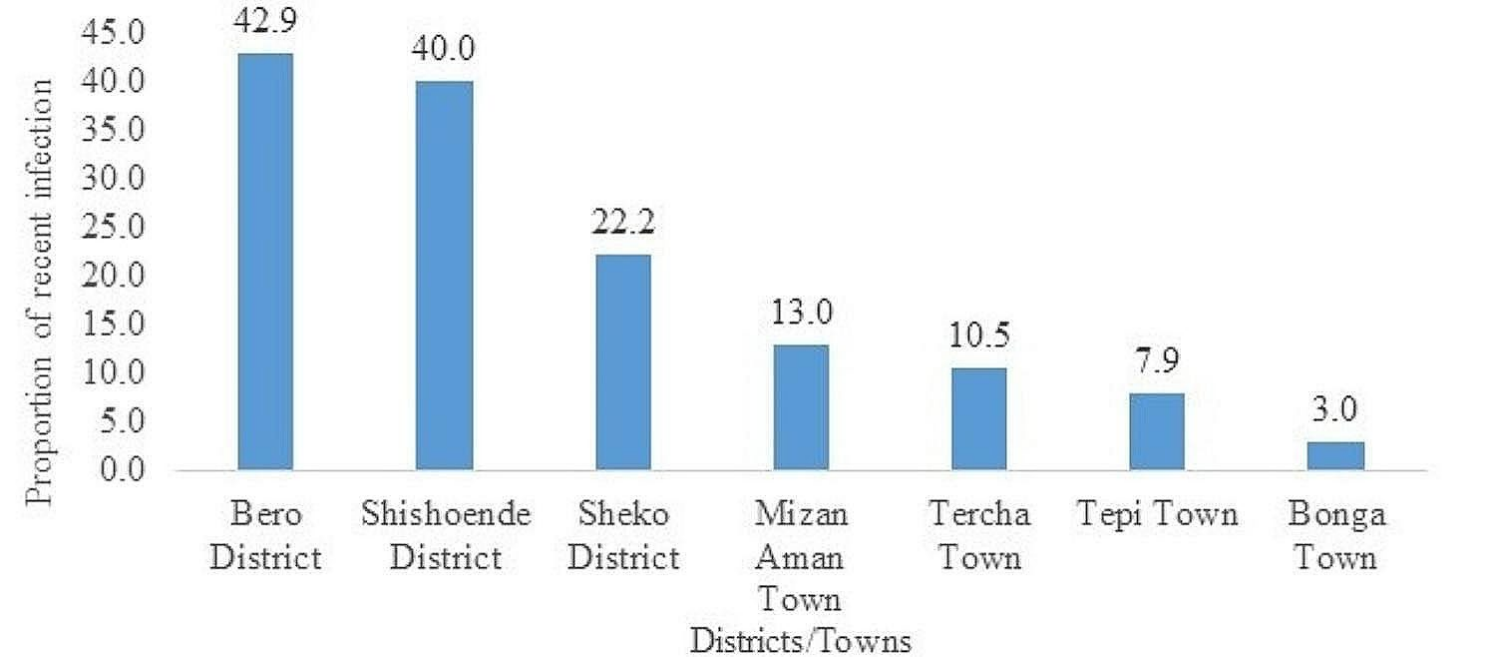
Proportion of recent infections among newly diagnosed HIV cases by districts/towns, Southwest Ethiopia Regional State, 2022

From the 786 individuals with valid recency test results, 89 (11.3%, 95% CI: 11.2, 11.5%) were recently infected with HIV. The proportion of recent HIV infections varied significantly across zones and geographic areas (Fig. 2), ranging from 42.9% in West Omo zone to 13.2% in Bench Sheko zone (Figs. 2 and 3).

The highest proportions of recent infections were observed among clients who were illicit drug users (23.1%), government/NGO employees (19.7%), and those aged 15–24 years (15.9%). Clients with a recent history of commercial sex practice had a 15.3% proportion of recent infection, while those with a recent history of invasive medical procedures had a 23.1% proportion. Similarly, the proportion of recent infections among prisoners were 14.4%, 9.6% in females, 12.9% in those who are in primary education (Table 1).

**Fig. 4:**
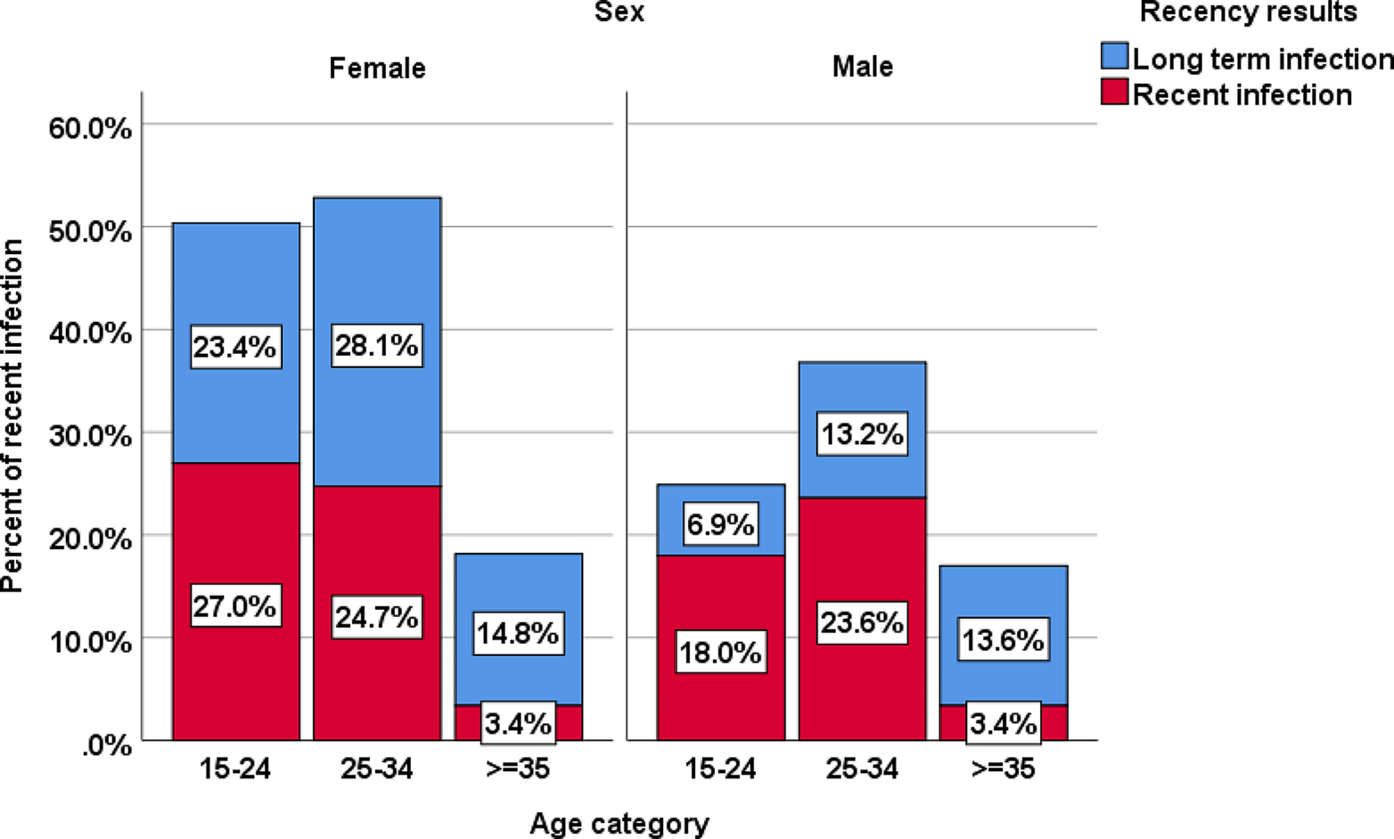
Recency test results among newly diagnosed HIV cases by age and sex, Southwest Ethiopia People Regional State, 2022

In the age group of 15–24 years, females accounted for 27% of recent infections, while males in the same age group accounted 18%. On the other hand, the proportion of long-term infections was higher in both males and females aged 35 years and older (Fig. 4).

Regarding HIV testing outlets, the majority, 30.2% of individuals with newly diagnosed HIV cases were reported from adult medical outpatient department and 21.9% from voluntary testing & counseling clinics (Table 2).

**Table 2:** Proportion of recent and Long-term infections by HIV testing outlets, Southwest Ethiopia Regional State, 2022

HIV testing outlets/point of care	HIV recent infection test result		Total (*N*/%)
	Long-Term (*N*/%)	Recent (*N*/%)	
Adult Medical Outpatient Department (OPD)	210 (88.6)	27(11.4)	237(30.2)
Antenatal, Labor & Delivery Clinic	87(90.6)	9(9.4)	96(12.2)
Antiretroviral Therapy Clinic	82(96.5)	3(3.5)	85(10.8)
Emergency OPD	68(89.5)	8(10.5)	76(9.7)
Key and Priority Population (i.e., commercial sex workers and prisoners)	90(80.4)	22(19.6)	112(14.2)
Tuberculosis and STI Clinic	3(37.5)	5(62.5)	8(1.0)
Voluntary Testing & Counseling Clinic	152(88.4)	20(11.6)	172(21.9)
Total	697(88.7)	89(11.3)	786(100.0)

### Trend of recent HIV infections among newly diagnosed HIV cases

**Fig. 5:**
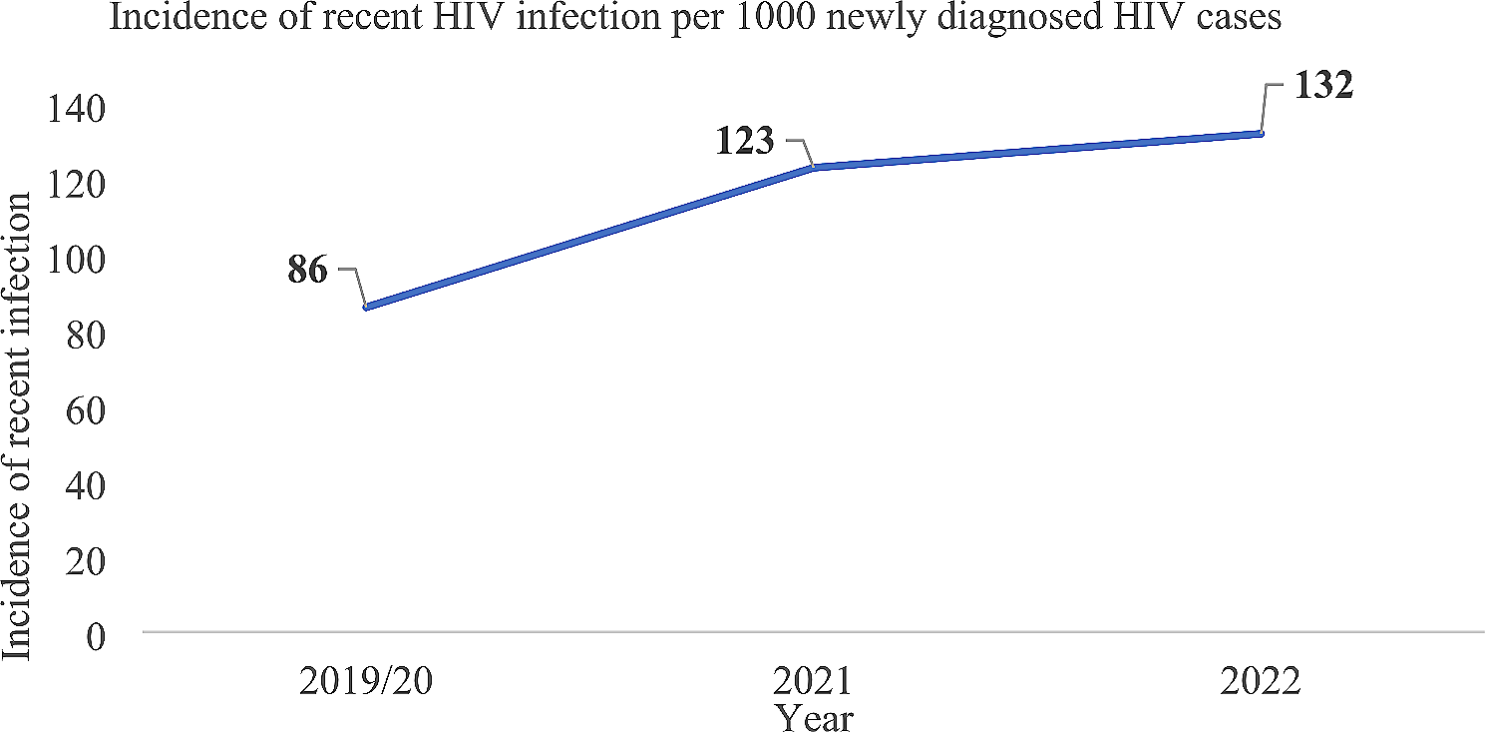
Trend of recent HIV infections among newly diagnosed HIV cases by incidence, Southwest Ethiopia Regional State, 2022

The overall incidence of recent HIV infections (for the three years) was 113 recent infections per 1,000 newly diagnosed HIV cases. The incidence is increasing from 86 (in 2019/20) to 132 (2022) recent HIV infections per 1,000 newly diagnosed HIV cases (Fig. 5).

### Risk factors associated with recent HIV infection

In the bivariate logistic regression analysis, variables with *p*-value < 0.25 were selected as candidate for multivariable logistic regression analysis. These includes age, sex, marital status, contact with index case, have you had sex in the last 12 months, involved in commercial sex practice in the last 12 months, and have you injected any illicit drugs to feel good or get high in the last 12 months. In the multivariable logistic regression analysis, recent HIV infection is significantly associated with variables such as young age, female sex, and contact history with index case (Table 3). The odds of recent HIV infections are seven times higher [AOR = 7.14, 95%CI: 2.89,17.57] in the age group 15–24 years and five times higher [AOR = 5.34, 95%CI: 2.20,12.94] in the age group 25–34 than their counterparts. Recent HIV infections are twice higher [AOR = 2.03, 95%CI: 1.26,3.25] likelihood of developing in the females than males. On the other hand, there is a 52% reduced [AOR = 0.48, 95%CI: 0.28, 0.83] risk of getting recent HIV infections in those who had contact history with the index cases than their counterparts (Table 3).

**Table 3:** Bivariate and multivariable logistic regression analysis for risk factors associated with recent HIV infections among newly diagnosed HIV cases, Southwest Ethiopia Regional State, 2021 (*N* = 786)

Study Variables	Category	Recency test results		COR (95% C.I.)	AOR (95% C.I.)	*P*-value
		Recent(N/%)	Long term(N/%)			
Age category	15–24	40	211	6.26(2.59,15.08)	7.14(2.89,17.57)	0.000
	25–34	43	288	4.93(2.06,11.79)	5.34(2.20,12.94)	0.000
	>=35	6	198	1	1	
Sex	Female	49	462	1.60(1.03,2.51)	2.03(1.26,3.25)	0.003
	Male	40	235	1	1	
Marital Status	Not married/cohabiting	30	176	1.48(0.89,2.46)	1.62(0.89,2.92)	0.111
	Divorced/widowed	19	173	0.95(0.54,1.69)	1.13(0.61,2.10)	0.701
	Married	40	348	1	1	
Contact with index case	Yes	60	552	0.54(0.34,0.88)	0.484(0.28,0.83)	0.008
	No	29	145	1	1	
In the last 12 months, have you had sex?	Yes	46	312	0.76(0.49,1.18)	0.87(0.53,1.43)	0.586
	No	43	385	1	1	
In the last 12 months, history of commercial sex practice? (Sex as main income or paid/received money or gift for sex)	Yes	22	122	0.65(0.38,1.09)	0.69(0.37,1.29)	0.246
	No	67	575	1	1	
In the last 12 months, have you injected any illicit drugs to feel good or get high	Yes	3	10	0.42(0.11,1.55)	0.49(0.11,2.16)	0.345
	No	86	687	1	1	

## Discussion

This HIV CBS analysis study is the second of its kind in Ethiopia, which focuse on recent HIV infections among newly diagnosed cases, providing valuable insights for a targeted and improved responses. The results will serve as a roadmap for individuals and organizations involved in various aspects of the HIV program, directing resources towards areas and population groups with a higher proportion of recent HIV infections. The study revealed that the overall incidence proportion of recent HIV infections in the Southwest Ethiopia Regional State was 11.3%. However, a significant portion of eligible cases (32.7%) did not undergo recency testing, highlighting the potential to impact the current findings of the study. Risk factors associated with recent HIV infections were being young individuals (15–24 and 25–34 years), females’ sex, and those who had history of contact with index cases.

The 11.3% finding in the present study is suggesting a slow progress towards the UNAIDS strategy of reducing new adult HIV infections to fewer than 200,000 by 2030. These results clearly indicate ongoing HIV transmission and prevalent risky behaviors in the study area [9]. The proportion of recent infections varies significantly across zones, with West Omo zone reporting 42.9% and Bench Sheko zone at 13.2%. This finding points to the presence of more transmission clusters and risky sexual networks in towns that serve as sources of infection. The study has identified priority geographic areas and sub-populations where HIV is actively transmitting. In line with the national HIV surveillance guideline, an enhanced response strategy is necessary to identify risky sexual networks and HIV transmission clusters [11].

In this study, the proportion of recent HIV infection was 11.3% (95% CI: 11.2, 11.5%), which is lower than those reported in many similar studies done on other countries. For instance, Germany reported a significantly higher proportion of 30.4% [20], Singapore at 19% [21], Uganda at 17% [22], Spain at 23% [23]and Northern Ethiopia at 14.2% [4]. However, the proportion found in this study is higher than those reported in Northern China (9.5%) [24], Kenya (8.6%) [25], Rwanda (6.1%) [5], and Malawi (3%) [26]. These differences in proportions can be attributed to several factors, including variations in HIV incidence rates across countries, differences in the recent infection testing algorithms used , and disparities in the total number of recent HIV infections diagnosed among newly diagnosed HIV cases. In addition, the discrepancy in the findings could be attributed to the lower registration of newly diagnosed HIV infections in the study area and decreased performance of recency testing. The study highlights the importance of improving the HIV case-based surveillance system to identify more recent infections. This would enable the implementation of more targeted prevention strategies to effectively control HIV transmission in the region.

The study also revealed variations in the proportion of recent HIV infections across different occupational types. A similar study conducted in Morocco found that commercial sex workers contributed to 14% of recent HIV infections, which is comparable to the present study finding of 13% [27]. This similarity might be due to the higher risky behaviors in these population groups, limited awareness on the HIV prevention and control measures, and large number of population movements to the urban areas.

Additionally, more than half (53%) of individuals with newly diagnosed HIV cases presented with WHO clinical stage III or IV at the time of recency testing. This finding suggests that a significant proportion of individuals were diagnosed with HIV at a late stage of disease progression, which can lead to decreased treatment outcomes and increased risk of HIV-related morbidity and mortality. Moreover, this study shows that 15.9% of recent infections were identified with advanced WHO HIV clinical staging (III/IV). This could be due to the gap in early HIV target testing in the health facilities and low awareness on the importance of HIV testing in the community. In Ethiopia, while the presence of illicit drug users is uncommon, this analysis uncovered a 1.7% proportion of this risky behavior in the region. This figure closely aligns with a study conducted in Northern Ethiopia, which reported 1.6% [4]. Hence, it is crucial to highlight and address these rare and emerging risky behaviors as they have the potential to accelerate the transmission of the virus.

The incidence of recent HIV infections increased from 2019/20 to 2022. This rise could be attributed to improvements in the reporting system at healthcare facilities during the implementation of the surveillance system. However, this contrasts with the Ethiopian National Strategic Plan for HIV 2021–2025 (NSP) which aimed to achieve HIV epidemic control by 2025 by reducing new HIV infections to less than 1 per 10,000 populations [28].

This study identified risk factors associated with recent HIV infections. The likelihood of recent HIV infections is significantly elevated in certain age groups, with seven times higher [AOR = 7.14, 95% CI: 2.89, 17.57] among individuals aged 15–24 years and five times higher [AOR = 5.34, 95% CI: 2.20, 12.94] among those aged 25–34 compared to their counterparts. This underscores the increased vulnerability to recent HIV infections in younger individuals, highlighting the importance of targeted interventions and awareness campaigns aimed at these age groups.

The study revealed that the majority of recent HIV infections were detected in younger age groups, 15.9% in 15–24 years and 13% in the 25–34 years, surpassing the findings of an HIV case-based surveillance study done at Kenya (9.6%) [29]. This rise in recent HIV infections among the younger population could potentially lead to a decline in national income productivity, as the disease burden impacts the economically active age group. This proportion is lower than the study done at Germany, which reported that 43% of recent infections were in the age group of 18-25years [20]. This discrepancy may be due to the larger study participants included in the later study. Additionally, this study reported that the proportion of recent HIV infections decreases as age increases, while long-term infections show the opposite trend. This is in alignment with a recency testing study conducted in Germany [20], which suggested that older age groups were more likely to have long-term HIV infections. The study done at Rwanda indicated that acquiring recent infections is reduced by 41.5% in the age group of 39–49 years [5]. The decrease in recent infections with age might be attributed to a reduced exposure to risky sexual behaviors among older individuals.

Recent HIV infections had twice higher [AOR = 2.03, 95%CI: 1.26,3.25] likelihood of diagnosis in females than males. This is consistent with reports from the WHO Africa regional office [30] and similar study in Uganda (AOR = 2.4) [22]. This increased risk may be attributed to engaging in risky sexual behaviors to cope with the rising socioeconomic burdens, facing anatomical risks, or experiencing minimal decision-making power concerning safe sex.

There is a 52% reduced opportunity of acquiring recent HIV infections for individuals with a history of contact with index cases compared to their counterparts [AOR = 0.48, 95% CI: 0.28, 0.83]. This finding aligns with research from Northern Ethiopia [4], which also indicated a 50% decrease in the likelihood of detecting recent HIV infection among clients with contact history with index cases. These results suggest that contact with an HIV index case acts as a protective factor against recent HIV infection. One possible explanation could be that contact with an index case may lead to increased HIV testing and earlier diagnosis, thereby reducing the window period for recent infection. Additionally, strategies such as index case testing and partner notification can aid in identifying and testing sexual partners, ultimately preventing new infections. Moreover, being aware of a partner’s HIV status can promote safer sexual practices and contribute to a reduction in HIV transmission. However, more research is needed to fully understand the mechanisms behind this association.

This study has strengths, primarily in its ability to identify recent HIV infections among newly diagnosed cases, which is a crucial first step in reducing further transmission within the community. However, there are some limitations that should be considered: Firstly, the lack of available viral load testing data prevents the reclassification of HIV infections as confirmed recent (for non-suppressed) or long-term (for virally suppressed) cases. Secondly, the findings represent the proportion of recent infections among newly diagnosed cases, which may not accurately reflect the actual HIV incidence in the general population. Despite these limitations, the identified risk factors associated with recent infections can provide valuable insights into the actual phenomena occurring in the source population.

### Conclusion and recommendations

This study indicated that the surveillance of recent HIV infections is a useful approach to monitor the HIV epidemic in Southwest Ethiopia Regional State. The finding shows a high proportion of recent HIV infections (11.3%) in the region. The risk factors associated with recent infections were young individuals, females, and those who had history of contact with index cases. Therefore, it is suggested that thelocal and regional health departments focus on areas with high proportion of recent infections by implementing improved HIV prevention strategies, particularly in hot spot areas to prevent further transmission. Ensuring the completeness of clinical data, including parameters like CD4 count, WHO HIV clinical staging, and viral load, is crucial for the precise reclassification of infections as recent or long-term. Moreover, to accurately calculate the true incidence of recent HIV infection in Ethiopia, it is essential to incorporate the total number of individuals tested for HIV into the HIV case-based surveillance system. By including this, a more comprehensive picture of HIV incidence rates can be obtained, enabling better-informed decision-making and targeted interventions to combat the spread of the virus effectively.

### Electronic supplementary material

Below is the link to the electronic supplementary material.

Supplementary Material 1

## Data Availability

The datasets used and/or analyzed during the current study are available from the corresponding author on reasonable request.

## Abbreviations

AIDS: Acquired Immune Deficiency Syndrome
AOR: Adjusted odds ratio
ART: Antiretroviral Therapy
CBS: Case-Based Surveillance
CD4: Cluster of Differentiation 4
CI: Confidence interval
CPAC: Case-Based surveillance data publication advisory committee
CRF: Case Report Form
EPHI: Ethiopian Public Health Institute
HIV: Human Immunodeficiency Virus
REDCap: Research Electronic Data Capture
SWERS: Southwest Ethiopia Regional State

## References

[1] UNAIDS, Global HIV, AIDS statistics Fact sheet. 2023. Available on: https://www.unaids.org/sites/default/files/media_asset/UNAIDS_FactSheet_en.pdf.; 2023.

[2] UNAIDS Data 2018. Accessed on Feb. 2022. https://www.unaids.org/sites/default/files/media_asset/unaids-data-2018_en.pdf.

[3] Mangal TD, Pascom ARP, Vesga JF, Meireles MV, Benzaken AS, Hallett TB (2019). Estimating HIV incidence from surveillance data indicates a second wave of infections in Brazil. Epidemics.

[4] Alemu T, Ayalew M, Haile M, Amsalu A, Ayal A, Wale F (2022). Recent HIV infection among newly diagnosed cases and associated factors in the Amhara regional state, Northern Ethiopia: HIV case surveillance data analysis (2019–2021). Front Public Health.

[5] Rwibasira GN, Malamba SS, Musengimana G, Nkunda RCM, Omolo J, Remera E (2021). Recent infections among individuals with a new HIV diagnosis in Rwanda, 2018–2020. PLoS ONE.

[6] Vesga JF, Cori A, van Sighem A, Hallett TB (2014). Estimating HIV incidence from case-report data: method and an application in Colombia. Aids.

[7] Dalal S, Johnson C, Fonner V, Kennedy CE, Siegfried N, Figueroa C (2017). Improving HIV test uptake and case finding with assisted partner notification services. Aids.

[8] Padilla M, Mattson CL, Scheer S, Udeagu CN, Buskin SE, Hughes AJ (2018). Locating people diagnosed with HIV for Public Health Action: utility of HIV Case Surveillance and Other Data sources. Public Health Rep.

[9] UNAIDS Issues New Fast-Track Strategy to, End AIDS. by 2030. Available online at:https://www.pedaids.org/2014/11/20/unaids-issues-new-fast-track-strategy-to-end-aids-by-2030/ (accessed May 28, 2022).

[10] Simon KR, Flick RJ, Kim MH, Sabelli RA, Tembo T, Phelps BR (2018). Family Testing: an Index Case Finding Strategy to close the gaps in Pediatric HIV diagnosis. J Acquir Immune Defic Syndr.

[11] EPHI. Guideline for HIV case based surveillance in Ethiopia. 2019.

[12] European Center for Disease Prevention and Control (2013). Monitoring recently acquired HIV infections in the European context.

[13] EPHI, HIV Case Based Surveillance. System in Ethiopia Guideline for Response to Newly Identified HIV Positive Cases. 2020.

[14] EPHI. Public Health Emergency Management Guideline for Ethiopia. Second ed. 2021.

[15] Using Recency Assays for HIV Surveillance: 2022 technical guidance. Geneva: Joint United Nations Programme on HIV/AIDS and the World Health Organization. 2022. Licence: CC BY-NC-SA 3.0 IGO.

[16] SEDIA Bioscience Corporation. Available online at:https://www.sediabio.com/point-of-care-resources/ (accessed May 16, 2024).

[17] Yufenyuy EL, Detorio M, Dobbs T, Patel HK, Jackson K, Vedapuri S (2022). Performance evaluation of the Asante Rapid Recency Assay for verification of HIV diagnosis and detection of recent HIV-1 infections: implications for epidemic control. PLOS Glob Public Health.

[18] Agyemang EA-O, Kim AA, Dobbs T, Zungu I, Payne D, Maher AD et al. Performance of a novel rapid test for recent HIV infection among newly-diagnosed pregnant adolescent girls and young women in four high-HIV-prevalence districts-Malawi, 2017–2018. (1932–6203 (Electronic)). 10.1371/journal.pone.0262071 PMC8836306 35148312

[19] CDC. Analyze and Interpret Surveillance Data. Facilitator Guide.2013.

[20] Hofmann A, Hauser A, Zimmermann R, Santos-Hövener C, Bätzing-Feigenbaum J, Wildner S (2017). Surveillance of recent HIV infections among newly diagnosed HIV cases in Germany between 2008 and 2014. BMC Infect Dis.

[21] Ang LW, Low C, Wong CS, Boudville IC, Toh M, Archuleta S et al. Epidemiological factors associated with recent HIV infection among newly-diagnosed cases in Singapore, 2013–2017. (1471–2458 (Electronic)). 10.1186/s12889-021-10478-5 PMC7927232 33653290

[22] Mermin J, Musinguzi J, Opio A, Kirungi W, Ekwaru JP, Hladik W (2008). Risk factors for recent HIV infection in Uganda. JAMA.

[23] Romero A, González V, Esteve A, Martró E, Matas L, Tural C (2011). Identification of recent HIV-1 infection among newly diagnosed cases in Catalonia, Spain (2006–08). Eur J Pub Health.

[24] Chen M, Ma Y, Chen H, Dai J, Luo H, Yang C (2019). Demographic characteristics and spatial clusters of recent HIV-1 infections among newly diagnosed HIV-1 cases in Yunnan, China, 2015. BMC Public Health.

[25] Welty S, Motoku J, Muriithi C, Rice B, de Wit M, Ashanda B et al. Brief Report: Recent HIV Infection Surveillance in Routine HIV Testing in Nairobi, Kenya: A Feasibility Study. (1944–7884 (Electronic)). 10.1097/QAI.0000000000002317 32058458

[26] Telford Ct Fau -, Tessema Z, Tessema Z, Fau - Msukwa M, Msukwa M, Fau - Arons MM. Arons Mm Fau - Theu J, Theu J Fau - Bangara FF, Bangara Ff Fau - Ernst A, Geospatial Transmission Hotspots of Recent HIV Infection - Malawi, October 2019-March 2020. (1545-861X (Electronic)). 10.15585/mmwr.mm7109a1 PMC8893337 35239633

[27] Mumtaz GR, Kouyoumjian SP, Hilmi N, Zidouh A, El Rhilani H, Alami K (2013). The distribution of new HIV infections by mode of exposure in Morocco. Sex Transm Infect.

[28] FHAPCO. HIV/AIDS National Strategic Plan for Ethiopia-2021-2025.

[29] Rice BD, de Wit M, Welty S, Risher K, Cowan FM, Murphy G (2020). Can HIV recent infection surveillance help us better understand where primary prevention efforts should be targeted? Results of three pilots integrating a recent infection testing algorithm into routine programme activities in Kenya and Zimbabwe. J Int AIDS Soc.

[30] HIV/AIDS (2017). Framework for action in the WHO African Region, 2016–2020.

