# Supplementary material for: Recent HIV infection and associated factors among newly diagnosed HIV cases in the Southwest Ethiopia Regional State: HIV case-based surveillance analysis (2019–2022)
Source: BMC Infect Dis. 2024 Jun 20;24:609. doi: 10.1186/s12879-024-09481-z (PMC11188228; doi:10.1186/s12879-024-09481-z)
Supplement: Supplementary file 1 — Supplementary Material 1 [file 12879_2024_9481_MOESM1_ESM.pdf]

JIMMA UNIVERSITY
ጅማ ዩኒቨርሲቲ

ቁጥር
Ref.No. EPID/473/2022
ቀን
Date 30/8/2022

To Ethiopia Public Health Institute (EPHI)-HIV CBS Case team/EPHI CPAC

Addis Ababa, Ethiopia

From Jimma University/Department of Epidemiology

Jimma, Ethiopia

Subject: **HIV Case Based Surveillance (CBS) Data Access Support Request**

We are writing this letter in support of our MPH field Epidemiology student Mr. **Nigatu Admsu (Cohort-VII, R-I HIV Track Resident)** for the acquisition of HIV secondary data from data base (REDCap) for his fulfilment for the requirement of Residency-I Output. Nigatu has been working as a residency-I (HIV Track resident) at Bonga Field Base and he want to analyse the South Western Regional (SWR) health bureau HIV case based Surveillance data as the requirement for his Residency-I output. He has completed a proposal related to HIV case Based Surveillance and has submitted to his mentors and advisor's for consideration. Currently, he is waiting your decision in access to the SWR health bureau data related to HIV Case Based Surveillance.

Since the time for residency-I is near completion, we would like to request your office (***EPHI HIV CBS Publication Advisory Committee (CPAC)***) to facilitate the usual cooperation according to your organizational data access policy in accessing the data for the resident.

Sincerely,

Dr. Lelisa Sena
Head, Department
of Epidemiology

የኢፒዲሚዮሎጂ ትምህርት ክፍል
Department of Epidemiology
College of Public Health & [illegible]
Jimma University

Tel. +251-47 11 114 57
PBX: +251471111458-60
Fax: +2514711114 50
+251471112040
P.O.Box. 378
JIMMA, ETHIOPIA
E-mail: ero@ju.edu.et
website: [illegible]

CamScanner
